# Evaluation of Changes in the Anthropometric Measurements of Infants in Relation to the Type of Feeding and the Presence of Gestational Diabetes in Their Mothers: A Preliminary Study

**DOI:** 10.3390/jcm14072393

**Published:** 2025-03-31

**Authors:** Dorota Ćwiek, Małgorzata Zimny, Weronika Dawid, Grażyna Iwanowicz-Palus, Bożena Kulesza-Brończyk, Kamila Rachubińska, Anna Maria Cybulska, Olimpia Sipak-Szmigiel, Dorota Branecka-Woźniak, Katarzyna Szymoniak

**Affiliations:** 1 Department of Obstetrics and Pathology of Pregnancy, Faculty of Health Sciences, Pomeranian Medical University in Szczecin, 71-210 Szczecin, Poland; dorota.cwiek@pum.edu.pl (D.Ć.); weronika.dawid@pum.edu.pl (W.D.); olimpia.sipak.szmigiel@pum.edu.pl (O.S.-S.); katarzyna.szymoniak@pum.edu.pl (K.S.); 2 Obstetrics Development, Faculty of Health Sciences, Medical University of Lublin, 20-081 Lublin, Poland; spupalus@gmail.com; 3 Department of Obstetrics, Gynaecology and Maternity Care, Faculty of Heatlh Sciences, Medical Univesity of Białystok, 15-089 Białystok, Poland; bozenabronczyk1@wp.pl; 4 Department of Nursing, Faculty of Health Sciences, Pomeranian Medical University in Szczecin, 71-210 Szczecin, Poland; kamila.rachubinska@pum.edu.pl (K.R.); anna.cybulska@pum.edu.pl (A.M.C.); 5 Department of Gynecology and Reproductive Health, Faculty of Health Sciences, Pomeranian Medical University in Szczecin, 71-210 Szczecin, Poland; dorota.branecka.wozniak@pum.edu.pl

**Keywords:** breastfeeding, gestational diabetes, infant growth, infant nutrition

## Abstract

**Background**: Breastfeeding is widely regarded as the optimal method of infant nutrition. A notable benefit of breastfeeding is its potential to avert the development of childhood overweight and obesity. This assertion holds particular significance in the context of infants whose mothers have exhibited gestational diabetes, a condition that has been demonstrated to be associated with an increased risk of carbohydrate and/or fat disorders in offspring, potentially leading to the onset of overweight and obesity in later life. **Objective**: The objective of the present study was to examine the variations in the anthropometric dimensions of infants across three distinct time points during the initial year of life, with a particular focus on the correlation between infant feeding practices and the prevalence of gestational diabetes in maternal subjects. Additionally, this study encompassed an analysis of the disparities in anthropometric dimensions between infant males and females. **Methods**: The study population included 42 infants whose mothers had been diagnosed with gestational diabetes between the 24th and 28th week of pregnancy, as well as 28 infants of women without gestational diabetes. The infants’ dietary habits, including breastfeeding, mixed feeding, and formula feeding, were assessed, and their anthropometric measurements were obtained at three time points: 7 ± 1 weeks postpartum, 6 months ± 1 week postpartum, and 12 months ± 1 week postpartum. The infants were measured for weight, length, head circumference, and thickness of the subscapular skin fold. We also calculated their BMI and Ponderal Index, and the measurements were referenced to WHO centile grids. **Results**: At 7 ± 1 weeks postpartum, exclusively breastfed infants exhibited higher weight compared to those who were mixed-fed or formula-fed (*p* = 0.03). However, at 1 year of age, breastfed infants demonstrated significantly lower weight compared to formula-fed infants (*p* = 0.019). Furthermore, at 12 months, breastfed boys exhibited lower weight, length, BMI, and lower subscapular skinfold thickness compared to formula-fed infants. **Conclusions**: Breastfeeding has been shown to play a pivotal role in preventing obesity in children. In the initial postnatal period, infants who are fed breast milk exhibit a higher weight compared to those who are fed formula. However, by the age of 12 months, the weight of breastfed infants typically falls below that of formula-fed infants. Diabetes during pregnancy has been observed to have no impact on the anthropometric dimensions of infants up to the age of one. Nevertheless, further research is necessary to comprehensively assess the long-term implications of maternal GDM in their offspring.

## 1. Introduction

It is a well-established fact that the first 2 years of a child’s life are a “critical period” due to rapid physical and mental development and metabolic programming. It is suspected that rapid weight gain during infancy and early childhood contributes to an increase in the number of subcutaneous fat cells and to the onset of overweight and obesity, the prevalence of which has emerged as a significant medical and social concern [1]. This phenomenon is attributable to the fact that obesity serves as a catalyst for the development of numerous diseases, including metabolic and oncological ailments. Consequently, it exerts a detrimental influence on the health of individuals, families, and society at large, given the substantial financial burden associated with treating these maladies. The failure to address this issue is likely to result in a continuous escalation in the proportion of overweight and obese individuals. A potential strategy to mitigate obesity might involve fostering the natural feeding of infants [2,3,4]. A substantial body of research has identified the pivotal role of breastfeeding in the developmental outcomes and the prevention of overweight and obesity in children. Infants who are fed breast milk typically consume at least 1.6 times less protein compared to those who are fed formula milk, and consequently, they are statistically less prone to developing obesity [5,6]. On the other hand, a high protein intake in formula-fed infants is believed to be associated with an increased risk of obesity, as it can lead to heightened lipogenesis and fat cell development, potentially through its impact on reducing human growth hormone production and fat breakdown [7]. This is associated with a higher risk of obesity in later life of about 20% [8]. A meta-analysis by Yan et al. found that the risk of obesity was 22% lower in children who had been breastfed compared to those who had not been given breast milk [4]. Moreover, a meta-regression conducted by Harder et al. demonstrated that the duration of breastfeeding was inversely associated with the risk of excess weight, with a 4% decrease in risk for each month of breastfeeding [9]. This phenomenon can be attributed to the weaker stimulation of growth hormone, insulin, and insulin-like growth factor 1 (IGF-1) by breast milk, resulting in the slower development of adipocytes in breastfed children [5]. Additionally, the presence of leptin in breast milk has been suggested as a potential contributor to the protective effects of breastfeeding on obesity, given its ability to suppress appetite and regulate caloric metabolism [4]. Consequently, the consumption of breast milk during infancy has been demonstrated to have a protective effect on the development of obesity in later life, as supported by numerous authors [10,11,12,13,14,15].

The period of fetal growth is also a critical period for an infant’s personal development, as it is when metabolic programming occurs. Maternal diseases, inadequate nutrition, or the use of stimulants can cause irreversible changes in the morphology, function, and metabolism of organs, which can affect children’s health in later years. Gestational diabetes and obesity in pregnant women play a special role [16]. Studies have shown that the risk of obesity, metabolic syndrome, type 2 diabetes, and impaired insulin sensitivity in the offspring of mothers with GDM (gestational diabetes mellitus) is two to eight times higher than in the offspring of mothers without GDM [17]. Furthermore, in a long-term study of the children of women with gestational diabetes, Nehring et al. demonstrated an association between maternal hyperglycemia and the development of obesity and impaired carbohydrate metabolism in childhood and adolescence, independent of maternal obesity and birth weight [18]. Gestational diabetes is also a risk factor for long-term cardiometabolic disease in the mother and offspring [19,20,21,22]. Therefore, it is imperative to maintain optimal glycemia levels to facilitate modification of fetal growth and, consequently, mitigate the risk of obesity and other adverse health outcomes in later years. However, it is crucial to acknowledge that obesity is influenced by a multitude of variables, including behavioral, genetic, and environmental factors [4], which underscore the necessity for a comprehensive approach to address this issue. Researchers have identified disparities in anthropometric measurements between male and female children, contingent on the method of breastfeeding or the existence of gestational diabetes in the mother [23,24,25,26].

In this aspect of nutritional programming, controlling infant growth is important. The benchmark is the developmental standards produced by the WHO in 2006. These standards were established using data collected in a multicenter growth reference study by the WHO. The growth standards include centiles and standard deviations (z-score system). The z-score system conveys the anthropometric value as the number of standard deviations from the median of the WHO reference population. The standards should be applied in each country and used to assess the growth of children [27,28,29].

Anthropometry is a rapid and non-invasive way to assess nutritional status. Body weight, length, and head circumference are the most common anthropometric measures used to gauge the nutritional status of newborns and infants. Birth weight is also used to classify newborns into appropriate or inappropriate weights for their gestational age [30,31] and later to evaluate growth. However, many authors believe that body weight alone is not an ideal parameter, as it does not reflect a person’s nutritional status and does not provide any information about fat distribution. A more authoritative measurement in infancy is skinfold thickness, which shows total and regional subcutaneous adipose tissue [32]. Therefore, skinfold measurements are more appropriate and provide meaningful information about the true volume of body fat in infants.

The significance of this article is predicated on the population under study, which consisted of infants postpartum to one year of age who were evaluated at four distinct time points during this period. A substantial number of anthropometric measurements were recorded for each participant, with the objective of ascertaining the correlation between breastfeeding and the incidence of diabetes in mothers during pregnancy. To the best of our knowledge, no comparable study has been conducted among the Polish population. A review of the literature reveals that, while studies have examined the relationship between infants’ anthropometry and breastfeeding or the onset of GDM, none have explored the interplay between these two factors.

Aim of this research: The objective of this study was to examine the variations in the anthropometric dimensions of infants across three distinct time points during the initial year of life, with a particular focus on the correlation between infant feeding practices and the prevalence of gestational diabetes in their mothers. Additionally, this study encompassed an analysis of the disparities in anthropometric dimensions between male and female infants.

## 2. Methods

This study was conducted from January 2017 to December 2019. The data are part of a larger study assessing the anthropometric dimensions of mothers and infants, the levels of selected hormones in maternal blood and breast milk, and the quality of women’s nutrition.

**Figure 1:**
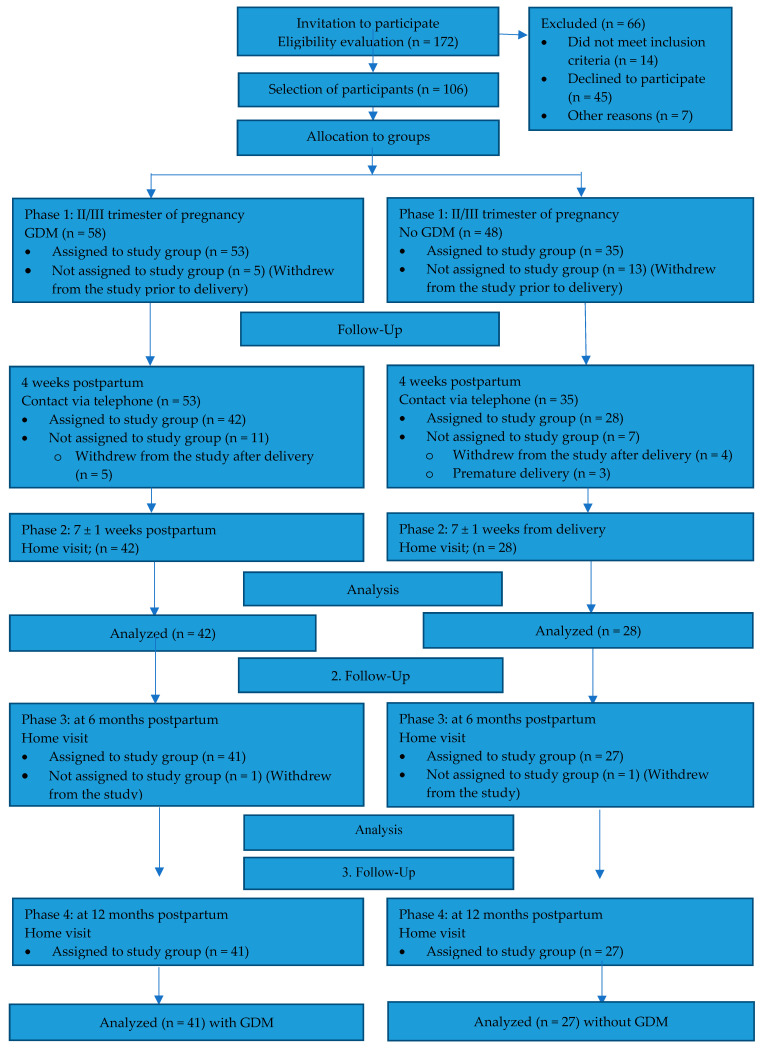
Study profile.

Population included in the survey: Our study involved 172 women in their second or third trimester of pregnancy (Phase 1) who were being monitored at the Outpatient Clinic for Women with Diabetes in Pregnancy and women attending follow-up examinations at the Outpatient Clinic for Pregnant Women at the University Clinical Hospital No. 1 in Police. Women who were pregnant and over the age of 18, with a singleton pregnancy, were invited to participate in the study. The inclusion criteria for the study group were a diagnosis of gestational diabetes based on an oral glucose tolerance test (OGTT) and the woman’s consent. The inclusion criteria for the control group were a normal OGTT result and the pregnant woman’s consent. The exclusion criteria for both groups were the onset of diabetes before pregnancy and delivery of the baby before the 37th week of pregnancy was completed. While still pregnant, 66 women were excluded from the study: 45 did not give their consent and 21 were not included for other reasons. Finally, 106 pregnant women who met the inclusion criteria were enrolled in the first phase. Subsequent to delivery, the exclusion criteria for both groups were modified to encompass births prior to the 37th week of pregnancy. Ultimately, all the criteria were found to have been satisfied by 42 women with gestational diabetes (study group) and 28 with no diabetes (control group) (60 women). The study proper was conducted 6–8 weeks postpartum (Phase 2). The subsequent meeting with the participants occurred six months postpartum (Phase 3). Unfortunately, 6 months after delivery, 1 patient in the GDM group and 1 patient in the non-GDM group withdrew from the study, so there were ultimately 41 women in the study group and 27 in the control group. The final meeting with the participants occurred one year after the initial enrollment, which corresponded to Phase 4 of the study. This phase entailed the administration of the same assessments employed in Phase 3 (Figure 1).

For the purpose of achieving the study’s objective, the infants under six months were also divided according to the type of nutrition they received: EBF—exclusive breastfeeding; MF—mixed feeding (feeding at least 4x per day with breast milk); NBF—non-breastfeeding (no breast milk given). After the 6th month, due to a need to supplement the diet and a lack of exclusive breastfeeding, the infants were divided into those who were breastfed (BF—breastfeeding) and those who were not (NBF—no breastfeeding).

The allocation of the women into the study and control groups was conducted in accordance with the results of the OGTT test. In Poland, the principles of universal screening for hyperglycemia in pregnancy, as well as the criteria for the diagnosis of such hyperglycemia, are in accordance with the guidelines established by the World Health Organization (WHO) [33].

Infants were also divided by sex to assess anthropometric differences between boys and girls in relation to breastfeeding and the incidence of GDM in the mother.

Study protocol. Phase 1. At the initial evaluation, the women’s height was measured using a standard medical scale with a height gauge. A structured interview was conducted, encompassing sociodemographic data such as age, educational attainment, place of residence, material status, labor force participation, and parenthood.

Phase 2. Six to eight weeks postpartum, a home visit was conducted, during which the birth records were examined for information regarding the infant’s sex, birth weight, and length. The birth weight and length were obtained from the infant’s health book. However, as the length was measured in the delivery room with a tape measure, starting from the infant’s fontanelle and following its curves to the end of the heel, the accuracy of these measurements was compromised. Consequently, they were not plotted on centile grids and should be considered approximate.

At six to eight weeks postpartum, the infants were examined for weight, length, and head circumference.

Phase 3. A subsequent examination was conducted approximately one week after the infants reached six months of age, with a margin of error of one week. The procedures involved included the measurement of weight, length, head circumference, and the thickness of subcutaneous adipose tissue located beneath the left shoulder blade. These measurements were comparable to those obtained during the initial examination.

Phase 4. The final examination was conducted when the infant was 12 months ± 1 week old. The examinations were performed by two trained individuals.

Measurement method. The infant’s weight was measured using a certified SECA electronic infant scale with an accuracy of 5 g. The infant was measured naked on a tared scale in the morning between 6:30 a.m. and 9 a.m. The infant’s length was measured with a SECA infantometer in the supine position to the nearest millimeter. The body mass index (BMI) was calculated as weight divided by length squared (BMI = weight/length^2^), and the Ponderal Index (PI) was also calculated as weight divided by length cubed (PI = weight/length^3^). Head circumference was measured with a SECA tape measure, with an accuracy of ±1 mm, at the height of the largest occipital protuberance and a point on the forehead between the frontal cusps. The thickness of subcutaneous adipose tissue was examined using a caliper under the left scapula, at a point directly under the apex of the lower angle of the scapula at an angle of 45° to the vertical, with an accuracy of 1 mm. A GMP/Holtain dial fold gauge with a measurement range of 0–45 mm and a measurement accuracy of 0.1–0.2 mm was used for measurement. The anthropometric protocol for each event necessitated at least two repetitions per measurement, and if the first two measurements differed by more than a predetermined threshold value (weight 20 g; length 5 mm; head circumference 3 mm), a third measurement was taken. The resulting measurements were recorded in the anthropometric protocol on an ongoing basis.

The results were subsequently plotted on WHO centile grids for both girls and boys, with the timing of the measurements taken at 7 weeks ± 1 week, 6 months ± 1 week, and 12 months ± 1 week taken into consideration. The WHO grids employed included weight for age, height for age, weight for height, BMI for age, head circumference for age, and subscapular fold thickness for age. However, as the centile grids for the thickness of subcutaneous adipose tissue under the shoulder only determine its parameters from the 3rd month onward, this measurement was not included in the 7 ± 1 week study. In the WHO centile grids, the z-score system expresses the anthropometric value as the number of standard deviations from the median of the WHO reference population [27,28,29].

Statistical analysis. The analysis was carried out in R, version 4.3.1 (R Core Team (2023); R: A language and environment for statistical computing; R Foundation for Statistical Computing, Vienna, Austria; URL https://www.R-project.org/ accessed on 12 September 2023), and using the licensed software Statistica 13.0 (StatSoft. Inc., Tulsa, OK, USA). The Shapiro–Wilk test was employed to evaluate the normality of the distribution of the variables under study. Within-group comparisons of the qualitative variable values (i.e., not expressed in number) were conducted using the chi-square test (with Yates adjustment for 2 × 2 tables) or Fisher’s exact test when low expected numbers were present in the tables. The Mann–Whitney test was employed to make comparisons of the quantitative variable values (i.e., expressed in number) in the two groups. When the quantitative variable values in three or more groups were compared, the Kruskal–Wallis test was used.

The analysis assumed a significance level of 0.05, so all *p*-values below 0.05 were interpreted as indicating statistically significant relationships. The analysis was carried out in R, version 4.3.1. R Core Team (2023). R: A language and environment for statistical computing. R Foundation for Statistical Computing, Vienna, Austria. URL https://www.R-project.org/ accessed on 12 September 2023.

The research protocol was approved by the Bioethics Committee of the Pomeranian Medical University in Szczecin (no. KB-0012/75/2015 of 22 June 2015 and KB-0012/61/2018 of 23 April 2018).

## 3. Results

The mean age among patients with gestational diabetes was 32.4 years, while the mean age among patients without diabetes was 30.85 years. The majority of the participants were married women (more than 70% in both groups), residing in urban environments (95.1% and 88.9%, respectively), with a satisfactory standard of living (61% and 74.1%, respectively), economically active (85.4% and 74.1%, respectively), and university educated (90.3% and 70.4%, respectively). The participants were more likely to be first-born (51.2% and 66.7%, respectively). The mean birth weight of the subjects was 3423.78 g in the gestational diabetes mellitus (GDM) group and 3372.22 g in the group without GDM. A statistically significant disparity was observed in the cesarean section delivery rate between the two groups, with 63.41% of women with GDM opting for this procedure, in contrast to 33.33% of those without GDM (*p* = 0.029). At 7 ± 1 weeks postpartum, the prevalence of breastfeeding among mothers in both groups was more than 50.0%, with a significant proportion (more than 30%) employing a combination method of feeding. At the 6-month mark, the breastfeeding rates among mothers with GDM and those without GDM were comparable (*p* > 0.05). However, by 1 year postpartum, the breastfeeding rate among subjects with GDM was significantly lower than among those without GDM (39.02% vs. 66.67%; *p* = 0.047). A subsequent statistical analysis revealed that the two groups were not statistically different in any of the analyzed sociodemographic variables. The complete data set is presented in Table 1.

Anthropometric data were measured at four time points (birth, 7 ± 1 weeks postpartum, 6 months postpartum, and 12 months postpartum) and are displayed in Table 2. No differences were observed between boys and girls in perinatal measurements. However, at 7 ± 1 weeks after delivery, boys exhibited significantly higher body weight, BMI, and head circumference. At the 6-month mark, a heightened body weight, length, and head circumference were observed in the male subjects, and this trend persisted at the 12-month evaluation (*p* < 0.05). The complete set of data is presented in Table 2.

Table 3 presents the impact of breastfeeding and non-breastfeeding on the anthropometric variables of infants at 7 ± 1 weeks postpartum, as well as at 6 and 12 months postpartum. The analysis revealed that infants who were exclusively breastfed at 7 ± 1 weeks postpartum exhibited a significantly higher weight compared to those who were mixed-fed or formula-fed (5055 g vs. 4795 g; *p* = 0.03). However, subsequent to the transfer of weight values to centile grids, no statistically significant differences were observed (*p* < 0.05). No further disparities were observed between the study groups during this period, similar to the findings at 6 months postpartum. However, by 1 year of age, breastfed infants exhibited a statistically significant weight reduction compared to formula-fed infants (9433 g vs. 10,055 g; *p* = 0.019). Additionally, disparities were identified in the centile grids that evaluate infant weight: for breastfed infants, the mean was 50.51 centiles, while for infants who received the formula, it was 66.16 centiles (*p* = 0.024). Statistical differences were evident in the BMI score (both index and centile; *p* = 0.015 and *p* = 0.014, respectively), Ponderal Index (*p* = 0.046), and length-to-weight ratio (*p* = 0.01).

The objective of this study was to ascertain whether the method of feeding exerts a differential impact on the anthropometric measurements of infants, contingent on their sex. During the 7-day period with a standard deviation of ±1 day, no statistically significant differences were observed between subjects of the same sex when the anthropometric data were examined. In accordance with the anticipated findings, during this period, male infants exhibited a significantly higher body weight and BMI (*p* = 0.001 and *p* = 0.06, respectively) and larger head circumference (*p* = 0.001) in comparison to female infants.

At six months postpartum, a significant difference in head circumference was observed between breastfed and formula-fed male infants (*p* = 0.02). This was the only observed difference between the infants of the same sex. In contrast, the discrepancy between male and female infants widened during this period. Female babies exhibited a significantly lower body weight compared to male babies (*p* = 0.001), a significantly shorter length (*p* = 0.002), and a significantly smaller head circumference (*p* = 0.001).

A substantial number of statistically significant differences were observed 12 months postpartum, exclusively in the male infants who had been breastfed and those who had not. No such differences were observed in the female infants. Consequently, the body weight of breastfed boys was found to be statistically lower than that of formula-fed boys, as indicated by both weight measurements and percentile grids (9626 g and 10,521 g; *p* = 0.005 and 46.67 percentile and 71.67 percentile; *p* = 0.005). A similar outcome was observed in boys with respect to BMI: 16.54 vs. 17.66; *p* = 0.01 and in percentiles 44.41 vs. 66.4; *p* = 0.012; with the length-to-weight ratio in the percentile grid (42 percentile vs. 69.1 percentile). The latter result was statistically significant (*p* = 0.004). Additionally, there was a statistically significant correlation between subscapular skinfold thickness, measured with calipers, and the BMI percentile (7.11 mm vs. 8.1 mm; *p* = 0.024 and 60.92 percentile vs. 80 percentile; *p* = 0.026, respectively). A noteworthy observation is that infants who were breastfed at 12 months exhibited higher subscapular skinfold thickness compared to those who were not breastfed. However, this difference did not reach statistical significance (8.32 mm vs. 7.22 mm; *p* > 0.05 and 74 percentile vs. 54.73 percentile; *p* > 0.05). It is possible that a larger sample size might have yielded more substantial differences. Conversely, a comparison of male and female infants at 12 months postpartum revealed significant disparities in weight (*p* = 0.003), length (*p* = 0.007), and head circumference (*p* = 0.001), with male infants exhibiting larger overall dimensions (Table 4).

The effect of maternal GDM on infant anthropometric measurements was also analyzed. No disparities in infant measurements were detected at any of the three designated time points (see Table 5).

The present investigation examined the hypothesis that diabetes during pregnancy exerts an influence on anthropometric measurements according to the child’s sex. No differences were observed between infants of the same sex; however, the expected differences between male and female infants were identified. At 7 ± 1 weeks postpartum, weight analysis revealed that boys of mothers with and without GDM had significantly higher body weight than girls (GDM: 5146 g vs. 4643 g; *p* < 0.05 and without GDM: 5233 g vs. 4633 g; *p* < 0.05, respectively). During this period, girls of mothers with GDM exhibited a significantly smaller head circumference compared to boys of mothers with GDM (36.8 cm vs. 37.9 cm; *p* < 0.05). However, this difference was not observed in infants of mothers without GDM.

At six months postpartum, male infants of mothers with and without GDM exhibited significantly higher weight, length, and head circumference compared to female infants (*p* < 0.05). At twelve months, only male infants of mothers without GDM demonstrated a significantly higher weight and body BMI compared to female infants (*p* < 0.05), with no such relationships observed in infants of mothers with GDM. However, boys of mothers with GDM exhibited significantly greater length than girls of mothers with GDM (*p* < 0.05), a finding that was not observed in the infants of mothers without GDM. Furthermore, head circumferences were significantly higher in male infants than in female infants of both mothers with and without GDM (*p* < 0.05).

At all three time points, no differences were observed between same-sex babies in mothers with and without GDM (Table 6).

## 4. Discussion

In this study, we examined the anthropometric dimensions of infants at four distinct time points: at birth, at 7 ± 1 weeks postpartum, at 6 months ± 1 week, and at 12 months ± 1 week postpartum. We also investigated potential differences between male and female infants. Our analysis focused on the impact of infant feeding methods and the presence of gestational diabetes during pregnancy on these measurements. Additionally, we explored the association of these factors with the infants’ sex. The findings of this study suggest potential strategies for increasing the prevalence of breastfeeding and for encouraging women with gestational diabetes mellitus (GDM) to maintain normal blood glucose levels during pregnancy, with the aim of preventing abnormal weight gain in their infants.

### 4.1. Change in Anthropometric Dimensions of the Infants Under Study at Birth and at Three Time Points

The mean weight of the infants in our study at 6 months of age was 7813.53 g, and at 12 months of age, the mean weight was 9738.97 g, which was comparable to the findings of Zimowski’s study at similar times (7.85 kg and 9.89 kg, respectively) [34].

The mean head circumference in our study was 37.44 cm at 7 ± 1 weeks and 42.59 cm at 6 months. In the study by Zimowski et al., slightly higher values were reported; at 5–7 months, the head circumference was 43.36 cm regardless of sex, and at 11–16 months, it was 46.47 cm [34]. In the group under study, the mean head circumference of infants at 12 months was 45.67 cm.

Schülter et al. noted that the thickness of the subscapular skinfold increased up to the infant’s third month of life, after which there was a continuous decrease in thickness up to the age of one year [1]. In our study, we also observed a decrease in the thickness of the subscapular skinfold in our sample at two measurements (6 and 12 months of age). Similar observations were made by Zimowski et al., who reported that the thickness of the subscapular skinfold in infants was 7.15 mm at 5–7 months and 6.75 mm at 11–16 months [34]. In contrast, our measurements at 6 and 12 months exhibited a mean subscapular skinfold thickness of 8.51 mm and 7.72 mm, respectively.

### 4.2. Effect of GDM on the Anthropometric Measurements of the Infants Studied at Three Time Points

The objective of this study was to assess the impact of gestational diabetes mellitus (GDM) in mothers during pregnancy on the anthropometric dimensions of their infants during their first year of life. The analysis revealed no statistically significant differences in any of the variables examined at the three time points of the study (see Table 5). It is well documented that infants of GDM mothers exhibit a higher birth weight [16,17,18], which may be associated with an increased risk of obesity in later life [35]. This is a grave concern, as obesity-related diseases are a primary cause of morbidity and mortality [36]. Our study revealed no significant differences in birth weight and length among newborns of mothers with and without gestational diabetes, suggesting potentially healthy glycemic control. Conversely, Silvermann et al. examined the infants of mothers with diabetes and found that, at birth, 50% of the newborns had a weight above the 90th percentile for gestational age. However, by 12 months of age, the median weight in this group was comparable to that of the reference population [23]. In the present study, a similar outcome was observed. At the 12-month mark, no statistically significant differences were identified in weight between infants of mothers with or without GDM (*p* > 0.05) (see Table 5). A number of studies have demonstrated significant heterogeneity in their results, particularly with regard to the impact of gestational diabetes mellitus (GDM) on infants’ anthropometric dimensions. This variability may be attributed to the diverse study methodologies employed and the influence of different dependent variables on the trait under investigation. A potential limitation of our study is its relatively brief duration of one year; in the future, the infants of mothers with GDM may exhibit higher BMIs, as reported by numerous authors [16,17,18].

### 4.3. Effect of GDM on the Anthropometric Measurements of the Infants Studied at Three Time Points, by Sex

We also examined the impact of gestational diabetes mellitus (GDM) on the anthropometric dimensions of infants according to their sex. The results indicated that at 7 ± 1 weeks postpartum, male infants exhibited higher weight compared to their female counterparts, irrespective of the maternal diagnosis of GDM (GDM: 5146.30 g vs. 4642.86 g; *p* < 0.05; without GDM: 5233.33 g vs. 4632.67 g; *p* < 0.05). A similar correlation was observed at 6 months; the male babies of mothers with and without GDM weighed significantly more than the girls (8123.70 g vs. 7402.86 g; *p* < 0.05; without GDM: 8313.33 g vs. 7238.67 g; *p* < 0.05). This outcome aligns with the broader literature, which consistently demonstrates that males tend to have higher birth weights than females. However, at 12 months postpartum, no significant differences in weight were observed between boys and girls in the group of mothers with GDM. In contrast, among mothers without GDM, boys exhibited significantly higher weights compared to girls (10,190.83 g vs. 9098.33 g; *p* < 0.05). In a similar vein, Ay et al. reported that, at 14 months, boys weighed significantly more than girls (10,839 g vs. 10,217 g; *p* < 0.05; Table 6) [24].

A subsequent analysis of the growth of infants born to mothers with and without GDM revealed no differences at birth or 7 ± 1 weeks after delivery between male and female infants. However, by 6 months, a significant difference was observed, with both groups of mothers demonstrating variations in infant growth. Significantly, a smaller body length was observed in girls (GDM: 65.25 cm vs. 67.91 cm; *p* < 0.05; without GDM: 65.25 cm vs. 68.88 cm; *p* < 0.05). Conversely, at 12 months, only the female infants of mothers with GDM exhibited a significantly lower length (74.29 cm vs. 76.76 cm; *p* < 0.05). However, no such difference was observed among the infants of mothers without GDM (*p* > 0.05) (Table 6). Other conclusions were reached by Silvermann et al., who observed no significant difference between the height of boys and girls of mothers with diabetes [23].

Sex-specific measurements of head circumference in the infants of mothers with and without GDM revealed that, across all three periods evaluated, male infants exhibited larger head circumferences compared to their female counterparts within both groups. However, at 7 ± 1 weeks after birth, no significant differences in head circumference were observed between male and female infants in the group of mothers without GDM. The potential explanations for these findings, which may be attributable to the limited sample size, are discussed in Table 6.

Analysis of BMI by child sex and maternal GDM status revealed that, at 12 months of age, boys in the group of infants born to mothers without GDM exhibited a significantly higher BMI compared to girls (17.9 vs. 16.05; *p* < 0.05) (Table 6). A study by Ay et al. found that the BMI of boys was higher than that of girls at 1.5 months after delivery, 6 months after delivery, and 14 months after delivery (*p* < 0.05) [24].

### 4.4. The Impact of Breastfeeding on the Anthropometric Measurements of the Babies Studied at Three Time Points

The present study analyzed the effect of breastfeeding on infants’ anthropometric measurements at three time points. The results demonstrated that, at seven weeks of age, exclusively breastfed infants exhibited significantly higher body weight compared to infants who were fed formula or a mixture of formula and breast milk (5055.38 vs. 4795.86; *p* < 0.05). However, at six months of age, the difference in body weight became undetectable. At 6 months of age, the mean body weight of formula-fed infants was 7742.0 g, while the mean body weight of artificially fed infants was 9422.65 g (*p* < 0.05). At 12 months of age, the mean body weight of artificially fed infants was 10,055.29 g (*p* < 0.05). Consistent findings were reported by Oddy et al., who noted that, at 52 weeks of age, formula-fed infants had a higher mean body weight (10,138 g vs. 9731 g, *p* = 0.041) [37]. This finding is corroborated by a subsequent study by Donma et al., which demonstrated that formula-fed infants exhibited a tendency toward lower weight during the initial 3 months (*p* < 0.05). During the subsequent 3 months, the weight gain observed in this group was significantly greater than that of breastfed infants [38]. Consistent findings, albeit over a more limited observation period, were reported by Ekelund et al., who observed a negative correlation between the extent of breastfeeding at 2.5 months and infant weight gain during the first 6 months [39]. Other authors have also found that breastfed infants exhibit faster growth during the first four months of life, yet by the age of 1 year, they tend to have a lower weight compared to formula-fed infants [40,41,42]. However, Oaklet et al. reported contrasting findings, noting that breastfed infants at 6 weeks of age exhibited comparable weight gain to formula-fed infants [25].

Oddy et al. found that, at 52 weeks of age, formula-fed infants were significantly longer than breastfed infants (75.6 cm vs. 73.7 cm, *p* = 0.011) [37]. However, this result is not supported by our study. At no time point were there significant differences in length between infants receiving breast milk and formula (*p* > 0.05). Conversely, Donma et al. reported comparable outcomes, concluding that, after the sixth month, there was no significant difference in length gain between infants fed formula and those fed breast milk [38].

A systematic review by Owen et al. found lower mean BMIs in breastfed infants in infancy compared to those fed artificially [43]. This finding aligns with the results of the present study, which found no differences in BMIs between infants studied at 7 ± 1 weeks. However, at 12 months, infants fed formula exhibited significantly higher BMIs compared to those fed breast milk (17.35 vs. 16.4; *p* = 0.015).

Chaudhary et al. found that the length-to-weight ratio was higher in subjects currently being breastfed [44]. In contrast, our study found that, at 7 ± 1 weeks, the ratio was higher for exclusively breastfed infants than for formula- or mixed-fed infants, although this difference was not statistically significant (52.78 vs. 44.93, respectively; *p* > 0.05). At 6 months, the rate was lower but not statistically significant between the study groups of infants. However, at 12 months, it was statistically higher for formula-fed infants (44.88 vs. 63.97; *p* < 0.01). A multiple regression analysis by Assunção et al. showed that exclusive breastfeeding for ≥6 months influenced lower mean z-scores of the weight-to-length ratio than for formula-fed babies [45].

### 4.5. The Impact of Breastfeeding on the Anthropometric Dimensions of the Babies Studied by Sex at Three Time Points

We also analyzed the effect of breastfeeding on anthropometric dimensions by sex at three time points. Our findings revealed that boys exhibited significantly higher body weight and head circumference compared to girls in each study. However, boys’ lengths were only higher at their 6th and 12th months, and the BMI was only higher at 7 ± 1 weeks. Concomitantly, Oaklet et al. reported that body weight during the initial six weeks of breast- or formula-fed infants was greater in boys than in girls (*p* < 0.05), a finding that aligns with our study (*p* < 0.001) [37]. Consistent with our findings, De Lucia Rolfe et al. reported that, at 12 months, boys exhibited statistically significant higher weight compared to girls (10.2 vs. 9.3; *p* < 0.05) and significantly greater length (76.4 vs. 74.9 cm; *p* < 0.05) [26]. Notably, at the 12-month mark, significant disparities emerged between breastfed and formula-fed boys with respect to weight, length, BMI, and subscapular fold thickness. However, no such differences were observed in any of the analyzed parameters between girls receiving breast milk and those who were formula-fed. This discrepancy might be attributed to the limited sample sizes in these groups. A study by Jaddoe et al. found that shorter breastfeeding was positively correlated with greater peripheral and total subcutaneous fat mass at 6 months (*p* < 0.05) but not at 24 months [46]. While the present study did not examine peripheral and total fat mass, a previous investigation by Jaddoe et al. revealed that, at 6 months, there were no differences in subcutaneous skinfold thickness among male infants based on the feeding method. However, by 12 months, formula-fed boys exhibited significantly greater subcutaneous skinfold thickness compared to breastfed boys (8.1 mm vs. 7.11 mm; *p* = 0.024).

The present study corroborates the findings of numerous researchers on the beneficial effects of breastfeeding on the weight of infants after one year of age and their health [5,8,9,10], which could serve as a rationale for breastfeeding at least until one year of age or longer. Given that 55% of Polish infants were breastfed for at least the first six months, and only 24% of children were breastfed at least until the age of 12 months [47], it is evident that the percentage of children fed with breast milk could be increased, which would translate into the health of children, mothers, and society. No similar study was found for the Polish infant population either.

### 4.6. Limitations

A potential limitation of this study is the homogeneity of the group, which consists of infants of the same ethnicity. Additionally, the small group size and the relatively brief study duration of one year may have influenced the findings.

## 5. Conclusions

Breastfeeding may contribute to the prevention of weight gain and obesity in children in the future. In the initial weeks following birth, infants who are fed breast milk exhibit higher body weights compared to those fed formula. However, by the 12th month, the body weights of breastfed infants are lower than those of infants fed formula. Further research is necessary to ascertain the long-term implications of maternal milk on health outcomes in children.

It has been observed that maternal diabetes during pregnancy does not affect the anthropometric dimensions of children up to age 1; however, further research is necessary to ascertain the long-term implications of maternal GDM on future generations.

## Figures and Tables

**Table 1:** Mothers’ sociodemographic and obstetric data.

Sociodemographic Data		All Group n = 68 (%)	GDM n = 41 (%)	No GDM n = 27 (%)	*p*
Marital status	Married	38 (55.88)	29 (70.73)	19 (70.37)	1
	Single	20 (29.41)	12 (29.27)	8 (29.63)	
Education	Vocational	2 (2.94)	1 (2.44)	1 (3.7)	0.073
	Secondary	10 (14.71)	3 (7.32)	7 (25.93)	
	Tertiary	56 (82.36)	37 (90.25)	19 (70.37)	
Place of residence	Rural	5 (7.36)	2 (4.88)	3 (11.11)	0.379
	Urban	63 (92.65)	39 (95.12)	24 (88.89)	
Income status	Medium	8 (11.76)	4 (9.76)	4 (14.82)	0.218
	High	45 (66.18)	25 (60.98)	20 (74.07)	
	Very high	15 (22.06)	12 (29.27)	3 (11.11)	
Occupational activity	Working	55 (80.88)	35 (85.37)	20 (74.07)	0.399
	Not working	13 (19.12)	6 (14.63)	7 (25.93)	
Birth rate	1	39 (57.36)	21 (51.22)	18 (66.67)	0.311
	2	20 (29.41)	15 (36.59)	5 (18.52)	
	3 and above	9 (13.24)	5 (12.19)	4 (14.82)	
Type of childbirth	Natural	33 (48.53)	15 (36.59)	18 (66.67)	0.029 *
	Surgical	35 (51.47)	26 (63.41)	9 (33.33)	
Type of feeding at 7 ± 1 week	Breastfeeding	39 (57.36)	23 (56,1)	16 (59.26)	0.874
	Combination	22 (32.36)	13 (31.7)	9 (33.33)	
	No breastfeeding	7 (10.29)	5 (12.19)	2 (7.4)	
Type of feeding at 6 months *	Breastfeeding	45 (66.18)	25 (60.98)	20 (74.07)	0.392
	No breastfeeding	23 (33.82)	16 (39.02)	7 (25.93)	
Type of feeding at 1 year *	Breastfeeding	34 (50.0)	16 (39.02)	18 (66.67)	0.047 *
	No breastfeeding	34 (50.0)	25 (60.98)	9 (33.33)	
Infant’s sex	Male	39 (57.36)	27 (65.85)	12 (44.44)	0.135
	Female	29 (42.65)	14 (34.15)	15 (55.56)	
Sociodemographic data		average	Average	average	*p*
Mother’s age	average	31.76	32.37	30.85	0.655

Key: n—number of patients, GDM—gestational diabetes mellitus, p *—statistical significance.

**Table 2:** Babies’ anthropometric data by sex.

Babies’ Anthropometric Data			Total Group n = 68	Boys n = 39	Girls n = 29	*p*
			Mean	Mean	Mean	
**Delivery**	Weight [g]	mean	3403.31	3452.56	3337.07	0.249
	Length [cm]	mean	54.24	54.33	54.1	0.915
	Ponderal Index [kg/m^3^]	mean	21.4	21.6	21.1	0.275
**at 7 ± 1 week**	Weight [g]	mean	4944.71	5173.08	4637.59	<0.001 *
	Length [cm]	mean	56.41	56.87	55.79	0.116
	BMI [kg/m^2^]	mean	15.55	16.01	14.92	0.006 *
	Head circumference [cm]	mean	37.44	37.88	36.85	0.001 *
	Ponderal Index [kg/m^3^]	mean	27.7	28.3	26.7	0.1
**at 6 months**	Weight [g]	mean	7813.53	8182.05	7317.93	<0.001 *
	Length [cm]	mean	66.94	68.21	65.23	0.002 *
	BMI [kg/m^2^]	mean	17.49	17.68	17.23	0.348
	Head circumference [cm]	mean	42.59	43.1	41.9	<0.001 *
	Ponderal Index [kg/m^3^]	mean	26.3	26.1	26.5	0.786
	Subscapular skinfold thickness [mm]	mean	8.51	8.53	8.49	0.936
**at 12 months**	Weight [g]	mean	9738.97	10,108.21	9242.41	0.003 *
	Length [cm]	mean	75.93	76.81	74.76	0.007 *
	BMI [kg/m^2^]	mean	16.87	17.14	16.51	0.094
	Head circumference [cm]	mean	45.67	46.24	44.9	0.001 *
	Ponderal Index [kg/m^3^]	mean	22.3	22.4	22.1	0.522
	Subscapular skinfold thickness [mm]	mean	7.72	7.64	7.83	0.896

Key: n—number of patients, BMI—body mass index; p *—statistical significance.

**Table 3:** Effect of feeding type on infant anthropometric variables at the 3 time points studied.

Variable	At 7 ± 1 Weeks Postpartum						*p*	6 Months Postpartum						*p*	12 Months Postpartum						*p*
	Breastfeeding			No Breastfeeding + Mixed Feeding				Breastfeeding			No Breastfeeding				Breastfeeding			No Breastfeeding			
	Mean	Min–Max	SD	Mean	Min–Max	SD		Mean	Min–Max	SD	Mean	Min–Max	SD		Mean	Min–Max	SD	Mean	Min–Max	SD	
weight [g]	5055.38	3660–6490	609.72	4795.86	3740–6070	481.24	0.03 *	7742.0	5930–10,370	836.77	7953.48	5700–9890	1072.48	0.361	9422.65	7070–12,390	1074.55	10,055.29	7350–12,460	1212.62	0.019 *
weight [percentile]	52.99	1–96	24.89	43.79	4–88.5	23.84	0.115	52.00	4–100	24.57	56.43	3–97.5	29.51	0.525	50.51	2.5–99	26.88	66.16	6–99	27.56	0.024 *
Length [cm]	56.50	51–61	2.63	56.29	52–61.5	2.73	0.526	66.70	58.5–78	3.64	67.41	60–79	4.57	0.599	75.78	68–80	2.76	76.09	70–82	2.92	0.863
length [percentile]	50.64	0–98,5	34.10	48.34	3–100	35.91	0.921	48.13	0–100	32.04	50.57	0–100	36.03	0.815	60.62	1–97	30.91	59.41	7–100	27.08	0.878
head circumference [cm]	37.4	34–40	1.32	37.5	35–40	1.20	0.935	42.39	39.5–44.7	1.16	42.99	39.5–45.5	1.40	0.065	45.42	42.5–48	1.32	45.93	42–50	1.80	0.144
head circumference [percentile]	34.01	0–97	25.96	39.34	1–90	28.93	0.434	40.11	1–96	25.55	48.78	2–92	25.39	0.203	47.65	4–99	28.72	57.00	2–100	32.03	0.186
BMI [kg/m^2^]	15.83	12.88–18.96	1.59	15.17	12.82–19.22	1.51	0.102	17.45	12.85–21.78	1.84	17.57	12.33–21.71	2.24	0.589	16.40	13.78–19.85	1.58	17.35	13.83–20.53	1.69	0.015 *
BMI [percentile]	52.87	4.5–97	31.23	40.74	1–100	30.12	0.135	53.93	0–100	30.86	57.13	0–100	34.57	0.618	42.97	1–98	31.26	61.93	0–99	29.17	0.014 *
Length/weight [percentile]	52.78	0–100	35.46	44.93	0–100	36.85	0.324	56.73	0–100	30.87	60.22	0–100	33.55	0.582	44.88	2–98	30.09	63.97	2–99	29.04	0.01 *
Ponderal Index [kg/m^3^]	28.1	21.6–35.4	0.33	27.1	20.9–35.3	0.35	0.229	26.3	16.5–34.2	0.36	26.3	18.0–33.5	0.43	0.837	21.7	17.8–25.9	0.23	22.8	17.2–27.4	0.24	0.046 *
Subscapular skinfold thickness [mm]	-	-	-	-	-	-	-	8.61	5–13	1.86	8.33	4.2–11	1.54	0.871	7.68	4.5–14	1.91	7.76	5–12.2	1.68	0.735
Subscapular skinfold thickness [percentile]	-	-	-	-	-	-	-	71.08	1.5–100	29.09	72.22	0–98.5	30.05	0.953	67.07	2–100	28.90	70.34	7–100	28.01	0.713

Key: BMI—body mass index, SD—standard deviation, Min—minimum, Max—maximum, p *—statistical significance.

**Table 4:** Effect of feeding type on infant anthropometric variables at 3 time points by infants’ sex.

Variable	Sex	At 7 ± 1 Weeks Postpartum			*p* B/G	at 6 Months Postpartum			*p* B/G	at 12 Months Postpartum			*p* B/G
		Breastfeeding	Mixed Feeding + No Breastfeeding	*p* B/B G/G		Breastfeeding	No Breastfeeding	*p* B/B G/G		Breastfeeding	No Breastfeeding	*p* B/B G/G	
		Mean	Mean			Mean	Mean			Mean	Mean		
weight [g]	B	5280	5002	0.065	0.001 *	8053.75	8387.33	0.189	0.001 *	9626.11	10,521.43	0.005 *	0.003 *
	G	4696	4575	0.616		7385.71	7140	0.494		9193.75	9302.31	0.779	
weight [percentile]	B	55.71	44.63	0.149	0.365	51.9	63.2	0.194	0.315	46.67	71.67	0.005 *	0.664
	G	48.63	42.89	0.631		52.12	43.75	0.51		54.84	57.27	0.779	
length [cm]	B	56.94	56.77	0.675	0.116	68.04	68.47	0.965	0.002 *	76.33	77.21	0.245	0.007 *
	G	55.8	55.79	0.983		65.18	65.44	0.883		75.16	74.27	0.184	
length [percentile]	B	49.48	47.47	0.862	0.862	51.67	52.8	0.942	0.267	57.39	63.71	0.245	0.838
	G	52.5	49.29	1		44.1	46.38	1		64.25	52.46	0.184	
head circumference [cm]	B	37.83	37.97	0.793	0.001 *	42.76	43.65	0.02 *	0.001 *	45.81	46.62	0.064	0.001 *
	G	36.72	37.0	0.581		41.96	41.75	0.921		44.98	44.81	0.912	
head circumference [percentile]	B	34.46	40.53	0.623	0.651	36.17	52.8	0.06	0.623	44.72	60.86	0.064	0.867
	G	33.3	38.07	0.511		44.62	41.25	0.844		50.94	50.77	0.912	
BMI [kg/m^2^]	B	16.31	15.56	0.123	0.006 *	17.48	18.0	0.323	0.348	16.54	17.66	0.01 *	0.094
	G	15.08	14.75	0.715		17.41	16.75	0.582		16.25	16.84	0.449	
BMI [percentile]	B	58.73	42.87	0.17	0.126	51.54	61.5	0.319	0.814	41.69	66.4	0.012 *	0.406
	G	43.5	38.46	0.793		56.67	48.94	0.643		44.41	54.69	0.456	
length/weight [percentile]	B	59.06	47.13	0.378	0.135	53.83	64.87	0.225	0.955	42.0	69.1	0.004 *	0.523
	G	42.73	42.57	0.81		60.05	51.5	0.574		48.12	55.69	0.614	
Ponderal Index [kg/m^3^]	B	2.87	2.75	0.27	0.1	2.59	2.66	0.502	0.786	2.17	2.29	0.1	0.522
	G	27.1	26.6	0.747		26.8	25.7	0.518		21.6	22.7	0.423	
Subscapular skinfold thickness [mm]	B	-	-	-	-	8.51	8.55	0.582	0.936	7.11	8.1	0.024 *	0.896
	G	-	-	-	-	8.71	7.9	0.365		8.32	7.22	0.124	
Subscapular skinfold thickness [mm]	B	-	-	-	-	70.27	76.77	0.572	0.842	60.92	80.0	0.026 *	0.65
	G	-	-	-	-	72.0	63.69	0.365		74.0	54.73	0.124	

Key: B—boys, G—girls, BMI—body mass index, p *—statistical significance.

**Table 5:** Anthropometric measurements of the infants studied at 3 time points in relation to the presence of GDM markers.

Variable	At 7 ± 1 Weeks from Delivery						*p*	at 6 Months from Delivery						*p*	at 12 Months from Delivery						*p*
	GDM			Without GDM				GDM			Without GDM				GDM			Without GDM			
	Mean	Min–Max	SD	Mean	Min–Max	SD		Mean	Min–Max	SD	Mean	Min–Max	SD		Mean	Min–Max	SD	Mean	Min–Max	SD	
weight [g]	4974.39	3740–6490	546.18	4899.63	3660–6160	611.06	0.552	7877.56	5700–9890	891.27	7716.30	5930–10,370	972.62	0.164	9844.39	7350–11,600	1173.89	9578.89	7070–12,460	1195.58	0.229
weight [percentile]	49.06	4–96	24.34	49.07	1–88.5	25.69	0.9	54.15	3–97.5	26.75	52.52	4–100	25.84	0.693	59.62	6–97.5	30.09	56.39	2.5–99	25.34	0.647
length [cm]	56.54	51–61	2.62	56.22	51–61.5	2.74	0.669	67.00	60.5–78	3.55	66.86	58.5–79	4.57	0.702	75.91	70–82	2.88	75.96	68–80	2.79	0.85
length [percentile]	50.73	3–98.5	35.55	48.04	0–100	33.81	0.87	48.83	0–100	31.24	49.15	0–100	36.58	0.866	57.59	7–100	29.30	63.70	1–99	28.29	0.39
head circumference [cm]	37.51	35–40	1.21	37.34	34–40	1.35	0.604	42.70	39.5–45.5	1.34	42.43	39.5–45	1.15	0.327	45.76	42–50	1.67	45.54	42.5–49	1.47	0.436
head circumference [percentile]	35.82	1–97	26.66	37.00	0–90	28.47	0.87	42.49	1–96	26.13	43.89	2–92	25.35	0.715	52.17	2–100	32.10	52.56	4–99	28.65	1
BMI [kg/m^2^]	15.57	12.83–19.22	1.50	15.51	12.82–18.96	1.72	0.851	17.62	12.33–21.71	2.14	17.29	14.57–21.78	1.70	0.418	17.08	13.83–20.53	1.87	16.56	13.78–19.96	1.37	0.158
BMI [percentile]	47.28	2.5–100	30.24	48.33	1–97	32.99	0.94	57.2	0–100	33.18	51.7	1.5–100	30.28	0.463	55.57	0–99	34.64	47.70	3–98	25.88	0.265
Length/weight [percentile]	46.73	0–100	35.34	53.54	0–99	37.26	0.629	59.17	0–100	32.80	56.00	3–100	30.19	0.603	56.88	2–99	34.11	50.70	4–99	25.37	0.347
Ponderal Index [kg/m^3^]	27.6	21.6–35.4	0.33	27.7	20.9–33.3	0.36	0.773	26.4	16.5–34.2	0.40	26.1	19.0–33.5	0.36	0.584	22.5	17.2–27.4	0.27	21.8	18.1–25.3	0.18	0.256
Subscapular skinfold thickness [mm]	-	-	-	-	-	-	-	8.46	5–11.7	1.57	8.59	4.2–13	2.04	0.98	7.61	4.5–12.2	1.65	7.90	5–14	1.99	0.678
Subscapular skinfold thickness [percentile]	-	-	-	-	-	-	-	71.82	1.5–100	29.35	70.93	0–100	29.50	0.945	67.67	2–100	28.55	70.28	7–100	28.37	0.735

Key: GDM—gestational diabetes mellitus, BMI—body mass index, SD—standard deviation, Min—minimum, Max—maximum.

**Table 6:** Effect of the presence of gestational diabetes mellitus (GDM) on infants’ anthropometric variables at 3 time points by sex.

Variable	Sex	at 7 ± 1 Weeks after Delivery				*p* B/B G/G	at 6 Months after Delivery				*p* B/B G/G	at 12 Months after Delivery				*p* B/B G/G
		GDM		Without GDM			GDM		Without GDM			GDM		Without GDM		
		Mean	*p* B/G	Mean	*p* B/G		Mean	*p* B/G	Mean	*p* B/G		Mean	*p* B/G	Mean	*p* B/G	
weight [g]	B	5146.30	0.002 *	5233.33	0.021 *	0.879	8123.70	0.043 *	8313.33	0.007 *	0.915	10,071.48	0.151	10,190.83	0.015 *	1
	G	4642.86		4632.67		0.57	7402.86		7238.67		0.711	9406.43		9089.33		0.621
weight [percentile]	B	51.09	0.386	52.25	0.463	0.903	55.44	0.66	58.04	0.407	0.891	60.54	1	59.21	0.845	1
	G	45.14		46.53		0.777	51.64		48.10		0.694	57.86		54.13		0.621
length [cm]	B	56.94	0.181	56.71	0.525	0.891	67.91	0.017 *	68.88	0.043 *	0.583	76.76	0.021 *	76.92	0.134	0.783
	G	55.75		55.83		0.948	65.25		65.25		0.793	74.29		75.20		0.441
length [percentile]	B	51.24	1	43.00	0.643	0.648	49.67	0.762	57.58	0.251	0.647	59.33	0.6	64.08	0.981	0.783
	G	49.75		52.07		0.983	47.21		42.40		0.743	54.21		63.40		0.441
head circumference [cm]	B	37.89	0.005 *	37.85	0.16	0.736	43.13	0.003 *	43.04	0.03 *	0.518	46.24	0.021 *	46.25	0.022 *	0.951
	G	36.77		36.93		0.427	41.86		41.94		0.565	44.82		44.98		0.93
head circumference [percentile]	B	37.70	0.296	34.75	0.678	0.715	42.89	0.825	41.83	0.476	0.735	53.22	0.989	53.83	0.768	0.951
	G	32.18		38.80		0.483	41.71		45.53		0.691	50.14		51.53		0.93
BMI [kg/m^2^]	B	15.89	0.065	16.30	0.053	0.374	17.72	0.755	17.60	0.486	0.73	17.12	1	17.19	0.028 *	0.845
	G	14.96		14.88		0.847	17.43		17.05		0.621	17.01		16.05		0.354
BMI [percentile]	B	50.31	0.425	57.83	0.179	0.513	56.59	0.847	52.62	1	0.715	55.15	0.65	54.67	0.164	0.819
	G	41.43		40.73		0.879	58.36		50.97		0.527	56.39		42.13		0.348
Length/weight [percentile]	B	50.30	0.394	63.88	0.164	0.28	58.70	0.815	56.67	0.942	0.843	57.00	0.721	55.67	0.558	0.796
	G	39.86		45.27		0.896	60.07		55.47		0.585	56.64		46.73		0.458
Ponderal Index [kg/m^3^]	B	28.0	0.35	28.8	0.126	0.391	263	0.714	25.8	0.755	0.663	22.4	0.523	22.3	0.114	0.988
	G	27.0		26.8		0.983	26.8		26.3		0.78	22.9		21.4		0.217
Subscapular skinfold thickness [mm]	B	-	-	-	-	-	8.35	0.699	8.93	0.694	0.714	7.57	0.772	7.82	0.98	0.843
	G	-	-	-	-	-	8.67		8.32		0.677	7.69		7.96		0.827
Subscapular skinfold thickness [percentile]	B	-	-	-	-	-	71.26	0.934	76.17	0.623	0.725	70.00	0.64	73.88	0.714	0.831
	G	-	-	-	-	-	72.89		66.73		0.661	63.18		67.40		0.861

Key: B—boys, G—girls, GDM—gestational diabetes mellitus, BMI—body mass index, p *—statistical significance.

## Data Availability

The raw data supporting the conclusions of this article will be made available by the authors, without undue reservation.
